# The Generation and Control of Irregularly Shaped Perfect Vector Vortex Beams on Hybrid Poincaré Spheres Using All‐Dielectric Metasurfaces

**DOI:** 10.1002/nap2.70020

**Published:** 2026-01-29

**Authors:** Xiaojie Sun, Xuan Yang, Haixu Tao, Haiyang Song, Jingpei Cai, Jiaxuan Zou, Jiajing He, Jun Wang

**Affiliations:** ^1^ Department of Interdisciplinary Photonics Shanghai Institute of Optics and Fine Mechanics Chinese Academy of Sciences Shanghai China; ^2^ Center of Materials Science and Optoelectronics Engineering University of Chinese Academy of Sciences Beijing China; ^3^ Shanghai Tech University Shanghai China; ^4^ School of Microelectronics Shanghai University Shanghai China

**Keywords:** hybrid Poincaré sphere, irregular‐shaped perfect vector vortex beams, metasurface

## Abstract

Irregular‐shaped perfect vector vortex beams (IPVVBs), as a novel form of structured light for optical field manipulation, have attracted significant attention due to their combined properties of spatial vectorization, orbital angular momentum control, and polygonal symmetry. Compared with traditional circularly symmetric vortex beams, IPVVBs offer notable advantages in mode control and freedom expansion, providing new possibilities for high‐dimensional optical field encoding, optical manipulation, and information transmission. In this paper, using an all‐dielectric metasurface designed with pure geometric phases, we achieve the generation and spatial polarization control of integer and fractional order irregular‐shaped perfect vector vortex beams on the hybrid Poincaré sphere by introducing a cross‐phase distribution. The proposed IPVVBs exhibit unique topological properties and stable propagation characteristics. Moreover, the designed metasurface exhibits excellent broadband performance, enabling efficient generation of IPVVBs across multiple wavelengths. Furthermore, we experimentally demonstrate the edge imaging capability of IPVVBs, achieving a resolution of up to 3.1 μm. This work opens up new research directions in cutting‐edge fields such as modern optical imaging, microscopic manipulation, and quantum communication.

## Graphical abstract

Based on an all‐dielectric metasurface with cross‐phase modulation, we achieve the generation and polarization control of integers and fractional order irregular perfect vector vortex beams on the hybrid Poincaré sphere, opening a new avenue for optical field manipulation.

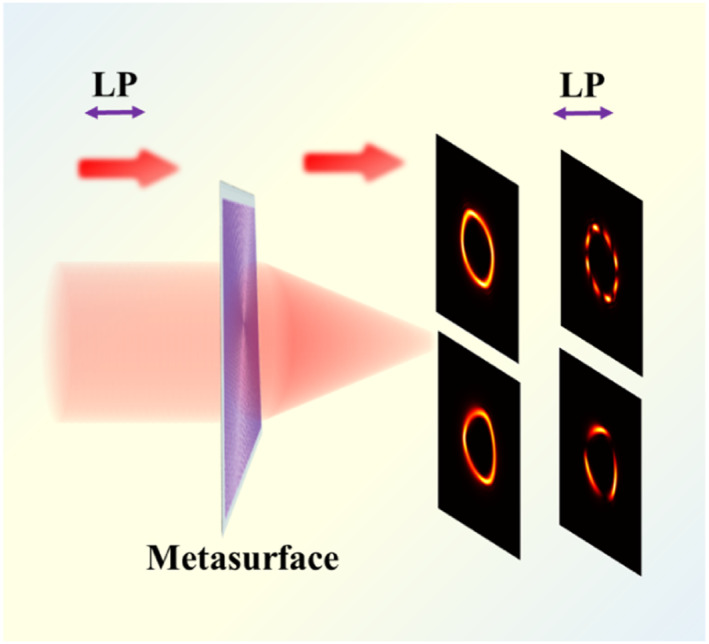

## 1 Introduction

The beam possesses multiple degrees of freedom such as polarization, amplitude, phase, and wavelength, which are fundamental properties that have attracted extensive attention in optical research. The conventional Poincaré sphere offers an intuitive geometric representation for polarization states, whereas its extended form—the hybrid Poincaré sphere—further integrates the degrees of freedom of polarization and spatial modes, serving as a crucial theoretical framework for describing and manipulating vector vortex beams [1]. The vector vortex beam is formed by the superposition of two vortices carrying opposite spin angular momentum (SAM) and orbital angular momentum (OAM) of *lћ*, where *l* denotes the topological charge [2, 3, 4]. The total angular momentum of a light beam comprises both SAM and OAM. Combining the characteristics of both vortex beams and vector beams, vector vortex beams exhibit broad application potential in areas such as optical imaging [5, 6], optical trapping [7, 8, 9], optical communications [10, 11], and quantum information processing [12, 13, 14]. Nevertheless, conventional vector vortex beams (VVBs) exhibit an inherent positive correlation between their transverse beam size and the magnitude of the carried orbital angular momentum (OAM). This correlation imposes significant limitations on their applications, particularly in constraining the resolution of optical microscopy, the channel capacity of optical communications [15], and the optical manipulation [16]. Therefore, perfect vector vortex beams have been proposed. However, the shape of perfect vortex beams is relatively singular; their intensity and polarization distributions mostly maintain regular, symmetric forms, lacking flexibility and making it difficult to adapt to more complex application scenarios. Unlike traditional circularly symmetric perfect vortex beams, irregular perfect vector vortex beams, while maintaining an annular energy distribution and stable topological structure, can have their intensity or polarization distributions designed into asymmetric, irregular, or even adaptive forms, enabling them to demonstrate greater potential in high‐dimensional information multiplexing, complex imaging, and topological optics research [17, 18].

The generation of perfect vector vortex beams has been explored through diverse technical approaches. The earliest implementations were mostly based on the Fourier transform of Bessel–Gaussian beams, combined with a spiral phase plate or an axicon to obtain a topological charge‐independent ring‐shaped spot [19, 20, 21]. Subsequently, researchers developed methods for sculpting the intensity morphology via composite phase modulation—such as high‐order cross‐phase or elliptized phase—enabling the generation of perfect vortex beams with polygonal or even irregular profiles in the Fourier plane [22, 23, 24]. Using spatial light modulators (SLMs) or digital micromirror devices (DMDs) also facilitates flexible control of beam intensity and the beam phase, allowing for the generation of programmable polygonal perfect vector vortices [25, 26]. Furthermore, methods based on diffractive optical elements (DOE) or modified spiral plates provide a stable passive component solution for polygonal vortex beams [27, 28]. Although these methods enable the generation of polygonal perfect vortices, they predominantly rely on complex optical setups or external programmable devices, often resulting in limited efficiency and compromised stability. In recent years, the development of all‐dielectric metasurface technology has opened up new opportunities in this field [29, 30, 31, 32, 33]. By designing geometric phase distributions at subwavelength scales, metasurfaces enable the simultaneous generation of high‐purity composite vortex beams and the manipulation of vortex arrays within single‐layer or multilayer planar devices [34, 35]. Expanding from scalar to vector beams, researchers have employed metasurfaces to introduce trigonometric functions, thereby generating tailored perfect vector vortex beams [36]. Additionally, designed metasurfaces have been utilized to directly produce perfect vector vortices in polygonal or irregular shapes [17]. Compared with traditional methods, metasurfaces not only possess advantages such as compact structure, high efficiency, broadband availability, and easy integration but also enable more complex optical field control [37, 38], including joint control of polarization state and topological state [39, 40, 41, 42]. By introducing irregularly shaped perfect vector vortex beams, it is possible to enhance the edges of complex objects without staining the samples. Moreover, due to its vector nature, it not only ensures the security of information but also enhances the reliability of the edge detection system. Therefore, exploring this cutting‐edge interdisciplinary field aims to break through the limitations of traditional optical components in terms of multifunctional integration and flexible control. By constructing this hybrid Poincaré sphere vector light field control platform, it is possible to provide a new solution for high‐contrast and super‐resolution microscopic imaging of complex samples.

In this work, we demonstrate the generation and manipulation of irregularly shaped perfect vector vortex beams (IPVVBs) on the hybrid Poincaré sphere using a single‐layer all‐dielectric geometric metasurface. These IPVVBs exhibit spatially tailored intensity distributions that are independent of both the polarization order and the spherical coordinates. By introducing cross‐phase modulation and superposing left‐hand and right‐hand circularly polarized beams carrying different topological charges, we generate integers and fractional order IPVVBs with controllable polarization orders. This approach enables optical field manipulation at arbitrary positions on the hybrid Poincaré sphere. The consistency between the simulated and experimental results has been verified. Finally, we employed the generated IPVVBs for edge imaging experiments, achieving a resolution of up to 3.1 μm. This metasurface platform for generating IPVVBs is anticipated to advance the miniaturization and on‐chip integration of optical systems, holding significant potential for future applications in optical microscopic imaging, biomedical science, quantum information processing, and related fields.

## 2 Result

### 2.1 Design of Metasurface to Generalized Irregular Perfect Vortex Beams on the Hybrid Poincaré Sphere

**FIGURE 1:**
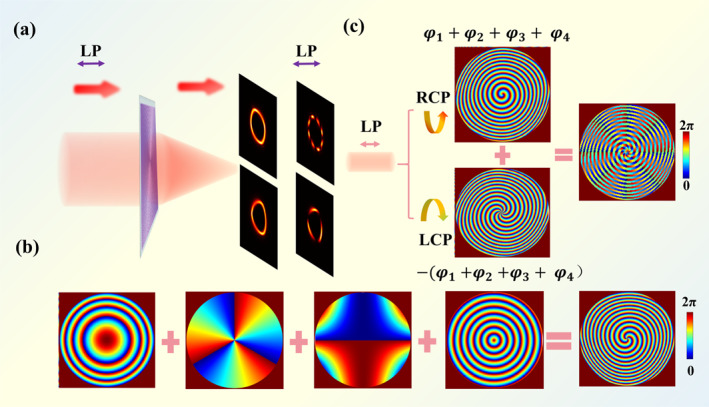
Design principle for generating IPVVBs based on an all‐dielectric metasurface. (a) Schematic of the generation of an IPVVB via an all‐dielectric metasurface. (b) Phase distribution for generating the IPVB. (c) Phase synthesis for constructing the IPVVB on the hybrid Poincaré sphere.

Figure 1a schematically illustrates the generation of an IPVVB from a linearly polarized Gaussian beam in free space, achieved by transmitting through an all‐dielectric metasurface with a specifically designed phase distribution. An IPVVB can be decomposed into two circularly polarized beams with opposite handedness and different absolute values of angular quantum numbers. The distribution of different polarization modes in such beams can be represented on the hybrid Poincaré sphere by (*σ, θ*). For two orthogonal circular polarization bases, the corresponding IPVVBs can be expressed on the hybrid Poincaré sphere as follows:

(1) $$|E_{\text{IPVVB}}\rangle =\left|A_{R}\right|e^{i\frac{\theta }{2}}|\text{IPVB},l_{m}\rangle +\left|A_{L}\right|e^{-i\frac{\theta }{2}}|\text{IPVB},l_{n}\rangle ,$$

Here, $|\text{IPVB},l_{m}\rangle$ and $|\text{IPVB},l_{n}\rangle$ denote the complex amplitudes of the IPVBs with right‐hand circular polarization (RCP) carrying a topological charge *l*_m_ and left‐hand circular polarization (LCP) carrying a topological charge *l*_*n*_, respectively. $\left|A_{R}\right|$ and $\left|A_{L}\right|$ denote the amplitudes of the RCP and LCP components, respectively, and *θ* represents the phase, where $\sigma \in \left[\frac{\pi }{2},\frac{\pi }{2}\right]$, θ∈[0,2π]. The polarization order *p* of the IPVVB represents the number of polarization state variations over one full rotation around the beam cross section, $p=\frac{l_{n}-l_{m}}{2}$, and the Pancharatnam topological charge is induced by the Pancharatnam–Berry (PB) phase, $l_{p}=\frac{l_{n}+l_{m}}{2}$.

To achieve the generation and manipulation of IPVVBs on the hybrid Poincaré sphere, it is necessary to first realize the generation of such irregular perfect vortex beams (IPVBs). Perfect vortex beams can be generated by applying a Fourier transform to a Bessel–Gaussian beam. Therefore, by simply introducing a Fourier transform lens to the generated Bessel–Gaussian beam and superimposing a cross‐phase, such beams can be effectively realized. The complex amplitude of such an IPVVB is expressed as follows:

(2) $$E_{\text{IPVVB}}=\frac{\omega_{0}}{\omega_{a}}\left[\exp\left(\frac{-{\left(\rho -\rho_{0}\right)}^{2}}{{\omega_{a}}^{2}}\right)\right]\times \left(\exp\left(i\varphi_{0}\right)\exp\left(i\varphi_{\text{IPVB}}\right)\left[\begin{array}{l} 1\\ i \end{array}\right]+\exp\left(i\varphi_{0}\right)\exp\left(-i\varphi_{\text{IPVB}}\right)\left[\begin{array}{l} 1\\ -i \end{array}\right]\right),$$

Here, $\frac{\omega_{0}}{\omega_{a}}\left[\exp\left(\frac{-{\left(\rho -\rho_{0}\right)}^{2}}{{\omega_{a}}^{2}}\right)\right]$ denotes the amplitude term, *ω*_*0*_ is the waist of the input Gaussian beam, and *ω*_*a*_ corresponds to the ring size of the generated perfect vortex beam. *φ*_*0*_ represents the initial phase, which is defined as zero. $\varphi_{\text{IPVB}}$ denotes the required phase profile to produce the IPVB, as illustrated in Figure 1b. Figure 1b shows a schematic of the phase distribution for generating an IPVB with the topological charge of 3. The phase expression for the IPVB is given by

(3) $$\varphi_{\text{IPVB}}=\varphi_{\text{lens}}+\varphi_{\text{spiral}}+\varphi_{\text{cp}}+\varphi_{\text{axicon}},$$

here,

$$\varphi_{\text{lens}}=\exp\left(i\frac{\pi \left(x^{2}+y^{2}\right)}{\lambda f}\right),$$

$$\varphi_{\text{sprial}}=\exp\left(il\varphi \right),$$

$$\varphi_{cp}=u\cdot x^{a}\cdot y^{b},$$

$$\varphi_{\text{axicon}}=\exp\left(-\frac{i\cdot 2\cdot \pi \cdot \sqrt{x^{2}+y^{2}}}{d}\right),$$

Here, *f* denotes the focal length of the metasurface in the Fourier transform lens phase term, and *λ* represents the wavelength of the incident light; *l* in the spiral phase term corresponds to the topological charge; in the cross phase, *u* controls the conversion efficiency of generating polygonal perfect vortex beams, while the exponents *a* and *b* are positive integers that determine the number of sides of the generated irregular shape; in the axicon phase term, *d* represents the period of the axicon, indicating the distance over which the radial phase changes by 2π. By adjusting parameter *d* in the axicon phase, the ring size can be effectively controlled; here, the value of the variable *d* remains unchanged and is 5 μm.

By superposing the phases shown in Figure 1b, only scalar IPVBs can be obtained. In this work, our control strategy is based on an all‐dielectric metasurface utilizing the geometric phase to achieve arbitrary polarization control on the hybrid Poincaré sphere. Because the geometric phase inherently exhibits spin locking, the “−” phase term is introduced in Equation (2) to break this intrinsic property, thereby enabling polarization control on the hybrid Poincaré sphere. As illustrated in Figure 1c, a linearly polarized light beam can be decomposed into left‐hand and right‐hand circularly polarized components. An irregularly shaped perfect vector vortex beam can be synthesized by coherently superposing two circularly polarized, irregularly shaped perfect vortex beams of opposite handedness, with topological charges of *l*_*m*_ and *l*_*n*,_ under the condition that $\left|l_{m}\right|\neq \left|l_{n}\right|$. The phase synthesis shown in Figure 1c demonstrates the method for generating a quadrilateral perfect vector vortex beam with a polarization order of 8. The detailed derivation of the geometric phase can be found in Supporting Information S1: Section 1. Accordingly, the phase expression for the IPVVB is given by

(4) $$\begin{array}{l} \varphi_{\text{IPVVB}}=\exp\left(i\left(\frac{\pi \left(x^{2}+y^{2}\right)}{\lambda f}+l\varphi +ux^{a}y^{b}-\frac{2\pi \sqrt{x^{2}+y^{2}}}{d}\right)\right)\\ +\exp\left(-i\left(\frac{\pi \left(x^{2}+y^{2}\right)}{\lambda f}+l\varphi +ux^{a}y^{b}-\frac{2\pi \sqrt{x^{2}+y^{2}}}{d}\right)\right) \end{array}.$$

**FIGURE 2:**
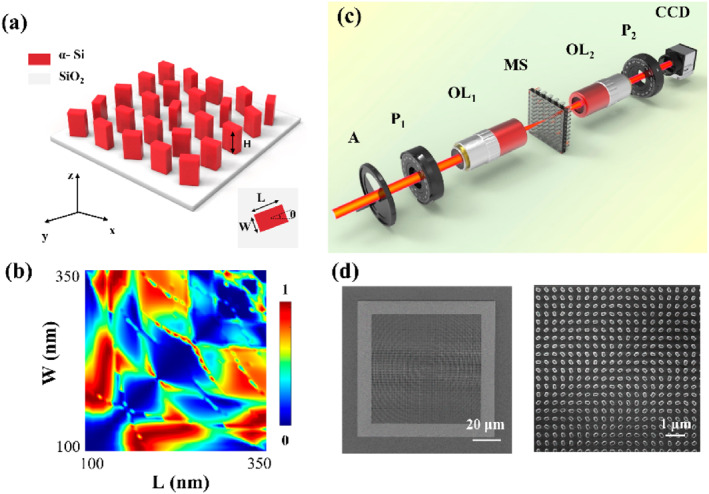
Design and characterization of the metasurface unit structures. (a) Schematic of the metasurface unit cell. (b) Polarization conversion efficiency as a function of unit cell size, varied from 100 to 350 nm. (c) Experimental setup for the generation and manipulation of IPVVBs on a hybrid Poincaré sphere. (d) SEM images of the amorphous silicon metasurface nanopillar array. A, attenuator; CCD, camera; MS, metasurface; OL_1_, objective lens; OL_2_, objective lens; P_1_, polarizer; P_2_, analyzer.

IPVBs with arbitrary polarization control on the hybrid Poincaré sphere are generated and manipulated using an all‐dielectric metasurface, as shown in Figure 2a. The basic structure of the metasurface consists of amorphous silicon (α‐Si) deposited on a fused silica substrate, with the substrate thickness being 500 μm and the α‐Si thickness 600 nm. Amorphous silicon has a higher refractive index of 3.73549 at a wavelength of 808 nm, and its absorption coefficient is 0, meaning there is no absorption. The period of the metasurface is 400 nm. We performed a parameter sweep of the nanostructure length and width from 100 to 350 nm with a step size of 5 nm. For the x‐polarized light incident structure, the relationship between the transmittance T_x_ and the phase distribution *φ*_*x*_ with respect to the length and width of the nanounit is shown in Supporting Information S1: Section 2. The polarization conversion efficiency of the structure is shown in Figure 2b. Based on the parameter sweep, the optimal structural dimensions were determined to be of length *L* = 195 nm and width *W* = 125 nm. The aspect ratio of the structure is at most 1:4.8. For actual processing, it poses lower difficulty. Furthermore, the designed structure exhibits a polarization conversion efficiency exceeding 95%. As shown in Supporting Information S1, the designed structure maintains high polarization conversion efficiency across a broad wavelength range from 760 to 880 nm. This is of great significance for the realization of multiwavelength multiplexing metasurfaces. It should be noted here that for a wavelength of 850 nm, the interaction between light and the structure resonates, so the resonance phenomenon can be found in the results of the polarization conversion efficiency. Figure 2c is the diagram of the transmission experimental setup we used to achieve irregular shaped perfect vector vortex beam control on the hybrid Poincaré sphere based on the designed all‐dielectric metasurface. A Gaussian beam emitted from a tunable laser first passes through a filter and a linear polarizer and is then focused onto the metasurface by the first objective lens (20×). After phase modulation by the metasurface, the beam is converted into the desired target optical field. The modulated beam is subsequently collected by the second objective lens (20×), where the polarization analyzer is used to characterize the polarization order of the vector beam, and finally, the beam is imaged using a CCD camera. Figure 2d shows the scanning electron microscopy (SEM) image of the fabricated metasurface. The left image displays a full view of the entire structure with a total size of 72 μm, whereas the right image presents a magnified version. The results demonstrate that the dimensional deviation between the fabricated nanostructures and the designed dimensions is controlled within 5%. Standard electron beam lithography is employed for the fabrication of metasurfaces. Because the fused silica substrate is nonconductive, a chromium layer was deposited to enhance the precision of the electron beam lithography. Otherwise, the fabricated metasurface unit structures would exhibit irregular shapes, posing significant challenges for experimental characterization. The detailed fabrication procedure is provided in Supporting Information S1: Section 6.

### 2.2 Generation of Irregular‐Shaped Perfect Vector Vortex Beams and Their Polarization Control on the Hybrid Poincaré Sphere via an All‐Dielectric Metasurface

**FIGURE 3:**
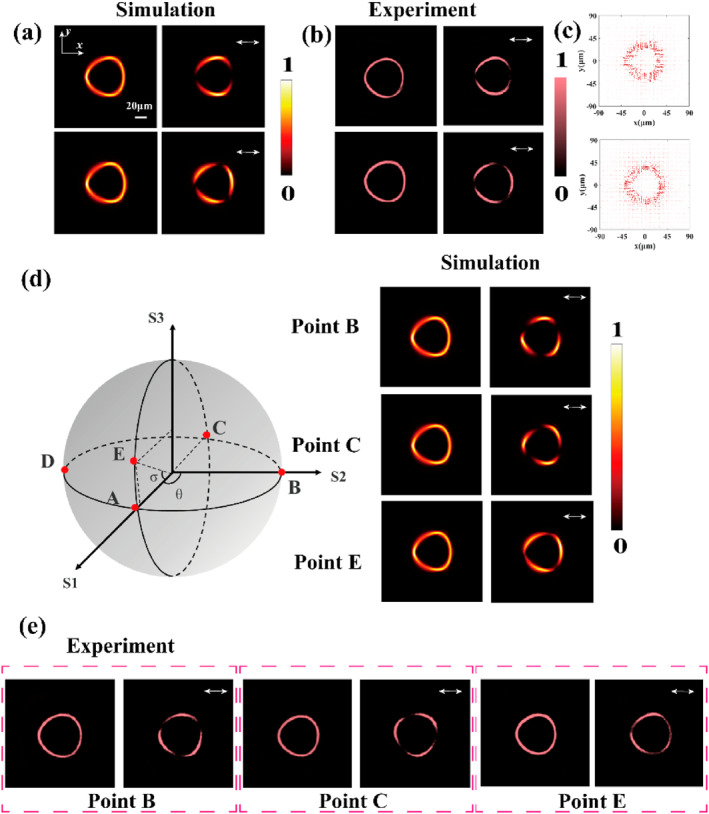
Generation and manipulation of triangular perfect vector vortex beams on the hybrid Poincaré sphere. (a) Numerically simulated intensity distributions on the x–y cross section for triangular perfect vector vortex beams with polarization orders *p* = 1 and *p* = 1.5. (b) Experimentally measured intensity distributions on the x–y cross section for beams with polarization orders *p* = 1 and *p* = 1.5. (c) Corresponding electric field vector distributions for triangular perfect vector vortex beams with polarization orders *p* = 1 and *p* = 1.5. (d) Numerically simulated intensity distributions on the x–y cross section at points B, C, and E of the hybrid Poincaré sphere. (e) Corresponding experimental results at points B, C, and E.

As a proof of concept, we first numerically and experimentally demonstrate the generation and polarization control of triangular perfect vector vortex beams on the hybrid Poincaré sphere and further investigate their integers and fractional order cases. The selected wavelength is 808 nm, the focal length is 200 μm, the structure size of the metasurface is 72 μm, and the numerical aperture NA is equal to 0.1. Based on the theoretical framework introduced in the previous subsection, generating a triangular beam requires to focus on controlling the cross‐phase parameters *u*, *a,* and *b*. The triangular beam profile can be achieved by setting *a* + *b* = 3. Accordingly, we select *a* = 1, *b* = 2, and *u* = 3 × 10^14^ for this study. For integer‐order and fractional order triangular perfect vector vortex beams, we study beams with polarization orders of 1 and 1.5, respectively. For a beam with polarization order *p* = 1.5, we achieve the control of arbitrary polarization on the hybrid Poincaré sphere, as shown in Figure 3. The first and second rows of Figure 3a present the simulated intensity distributions of beams with polarization orders of 1 and 1.5, respectively, obtained using the vectorial Rayleigh–Sommerfeld diffraction theory. These profiles correspond to triangular perfect vector vortex beams generated by superposing RCP and LCP triangular perfect vortex beams in the states $|\text{IPV}B_{R},0\rangle$ and $|\text{IPV}B_{L},2\rangle$ for integer order *p* = 1, and $|\text{IPV}B_{R},2\rangle$ and $|\text{IPV}B_{L},5\rangle$ for fractional order *p* = 1.5. The second row of Figure 3a corresponds to point A (0, 0) on the Poincaré sphere. Figure 3b presents the measured intensity distributions in the x–y cross section for both fractional and integer orders, captured by the CCD camera using the experimental setup illustrated in Figure 2c. The first column presents the intensity distributions of the linearly polarized Gaussian beam after transmission through the metasurface, whereas the second column shows the corresponding intensity distributions after passing through the analyzer. It can be observed that the beams split into two and three lobes, respectively, which correspond to twice the polarization order of the vector beams, that is, |2*p*|. Figure 3c presents the corresponding electric field vector distributions, further confirming the spatially inhomogeneous polarization characteristics of the beams. Because any point on the hybrid Poincaré sphere can represent the arbitrary perfect vector vortex beam, the longitude and latitude coordinates (*σ*, *θ*) on the sphere can be used to represent the polarization state relationship of the beam, with the geometric relation of $\theta =\arctan\frac{S_{2}}{S_{0}}=\phi$ and $\sigma =\arcsin\frac{S_{3}}{S_{0}}=\arcsin\left(\frac{{\left|A_{R}\right|}^{2}-{\left|A_{L}\right|}^{2}}{{\left|A_{R}\right|}^{2}+{\left|A_{L}\right|}^{2}}\right)$. Figure 3d shows the numerical simulation results corresponding to points B(0, π/2), C(0, π), and E(π/6, 0) on the Poincaré sphere, and Figure 3e shows the corresponding experimental results. A high level of agreement between the simulations and experiments can be observed. Notably, the shape and size of the triangular perfect vector vortex beams remain unchanged regardless of variations in the topological charge or polarization order. The polarization directions at points A, B, C, and E are observed to change accordingly. In addition, we investigate the numerical simulation results for different combinations of *a* and *b*. Varying these parameters alters the rotation direction of the triangular beams, as shown in Supporting Information S1: Figure S4. We also examine the influence of *u* on the results, as presented in Supporting Information S1: Figure S5, demonstrating that *u* plays a significant role in both the conversion efficiency and the control of the triangular curvature of the beams.

**FIGURE 4:**
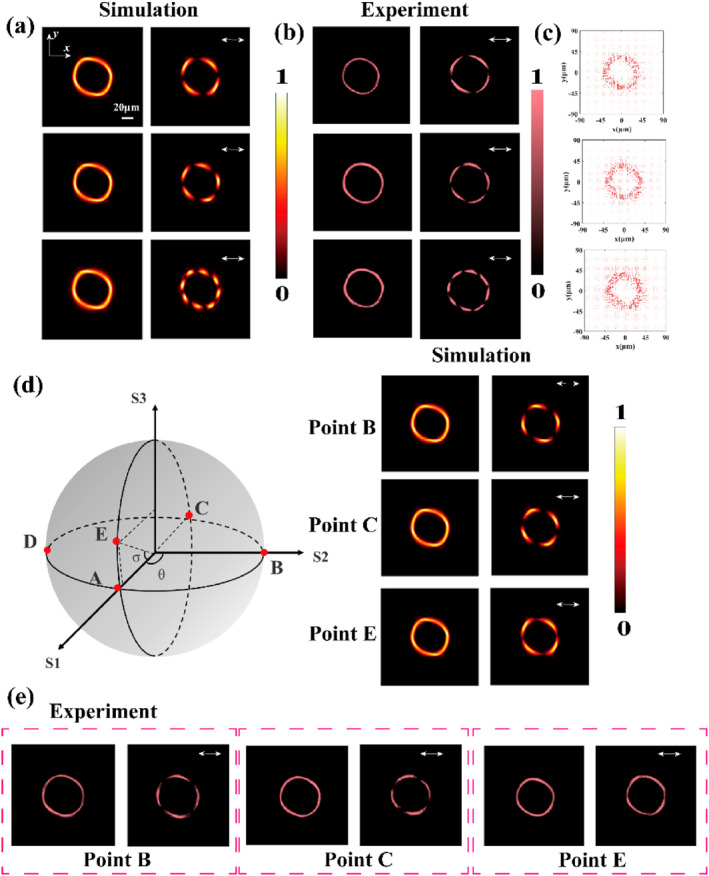
Generation and control of quadrilateral perfect vector vortex beams on the hybrid Poincare sphere. (a) Simulated intensity distributions on the x–y cross section of quadrilateral perfect vector vortex beams with polarization orders *p* = 2, *p* = 2.5, and *p* = 4. (b) Experimental intensity distributions on the x–y cross section of quadrilateral perfect vector vortex beams with polarization orders *p* = 2, *p* = 2.5, and *p* = 4. (c) Corresponding electric field vector distributions of quadrilateral perfect vector vortex beams with polarization orders *p* = 2, *p* = 2.5, and *p* = 4. (d) Simulated x–y cross‐sectional intensity distributions of quadrilateral perfect vector vortex beams at points B, C, and E on the Poincaré sphere. (e) Experimental results corresponding to the x–y cross‐sectional intensity distributions of quadrilateral perfect vector vortex beams at points B, C, and E on the Poincaré sphere.

Subsequently, we investigate the generation and polarization control of quadrilateral perfect vortex beams on the hybrid Poincaré sphere. Beams with integer polarization orders of *p* = 2 and *p* = 4, as well as the fractional order of *p* = 2.5, are studied, as shown in Figure 4. Similarly, the operating wavelength is selected as 808 nm, with the metasurface size of 72 μm and the focal length of *f* = 200 μm. For the generation of quadrilateral perfect vector vortex beams, according to the theoretical framework, the condition *a* + *b* = 4 must be satisfied. Therefore, we set *a* = 1 and *b* = 3. In addition, the parameter is set to *u* = 1 × 10^19^. Figure 4a presents the results calculated based on the vector Rayleigh–Sommerfeld diffraction theory. Each row corresponds to the generation of quadrilateral perfect vector vortex beams with polarization orders *p* = 2, *p* = 2.5, and *p* = 4, respectively. These beams are generated by the superposition of right‐hand and left‐hand quadrilateral perfect vortex beams with different topological charges, and their complex amplitudes are respectively represented by $|\text{IPV}B_{R},0\rangle$ and $|\text{IPV}B_{L},4\rangle$, $|\text{IPV}B_{R},-1\rangle$ and $|\text{IPV}B_{L},4\rangle$, $|\text{IPV}B_{R},-1\rangle$ and $|\text{IPV}B_{L},7\rangle$. The first column shows the total field distribution, whereas the second column presents the corresponding field distribution after passing through an analyzer. Figure 4b displays the experimental results of quadrilateral perfect vector vortex beams with different polarization orders. After passing through an x‐polarization analyzer, the beams are observed to split into four, five, and eight petals, respectively. The experimental results are in excellent agreement with the numerical simulations. Figure 4c presents the corresponding electric field vector distributions. Furthermore, we select the quadrilateral beam with the polarization order of *p* = 2 to investigate its polarization control on the Poincaré sphere. The result at point A (0, 0) on the hybrid Poincaré sphere is shown in the first row of Figure 4a and b. Points B, C, and E correspond to (0, π/2), (0, π), and (π/6, 0), respectively, on the Poincaré sphere, and their numerical simulation results and experimental measurements are shown in Figure 4d and e, respectively. The first column represents the total field distribution, whereas the second column shows the corresponding optical field distribution after passing through the analyzer. It can be observed that the lobes’ rotation direction of the quadrilateral perfect vector vortex beams changes after analyzer modulation, further confirming the spatial polarization variations at different positions. The experimental results correspond one to one with the numerical simulation results.

In summary, we have successfully generated triangular and quadrilateral perfect vector vortex beams using the cross‐phase design and achieved polarization control on the hybrid Poincaré sphere for selected polarization orders. For generating beams of other shapes, appropriate design of the parameters *u*, *a*, and *b* allows the production of both integers and fractional order vector beams by superposing left‐hand and right‐hand beams with different topological charges. Moreover, by manipulating the amplitude and phase distributions of IPVVBs and varying the position (*σ*, *θ*) on the sphere, spatially inhomogeneous polarization of the beams on the hybrid Poincaré sphere can be effectively manipulated. The diffraction efficiency of the designed metasurface is 43%.

### 2.3 Multiwavelength Control of Irregular‐Shaped Perfect Vector Vortex Beams

**FIGURE 5:**
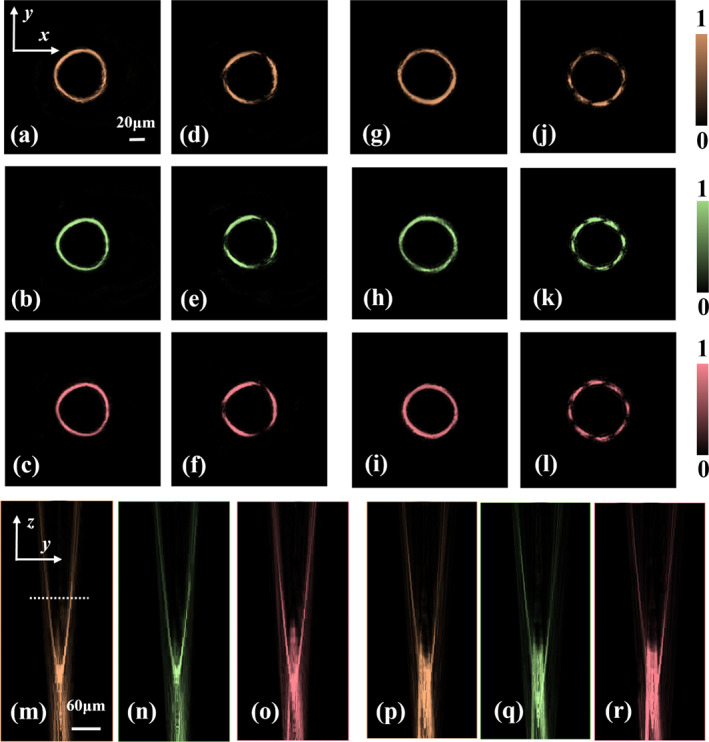
Multiwavelength multiplexing results of irregularly shaped perfect vector vortex beams. (a–c) Electric field intensity distributions in the x–y plane for triangular perfect vector vortex beams with polarization order *p* = 1.5 at wavelengths of 780 nm, 795 nm, and 808 nm, respectively. (d–f) Corresponding results for the triangular beams after passing through an analyzer. (g–i) Electric field intensity distributions in the x–y plane for quadrilateral perfect vector vortex beams with *p* = 4 at 780 nm, 795 nm, and 808 nm, respectively. (j–l) Corresponding results for the quadrilateral beams after passing through an analyzer. (m–r) Propagation field distributions in the y–z plane for the triangular and quadrilateral perfect vector vortex beams across the three wavelengths.

Here, we further investigate the wavelength‐multiplexing characteristics of irregularly shaped perfect vector vortex beams. As previously demonstrated in Supporting Information S1: Figure S2, the amorphous silicon nanounits maintain high polarization conversion efficiency across the wavelength range of 760–880 nm, indicating the broadband capability of the metasurface. Because Equation (3) in the theoretical section considers only single‐wavelength phase superposition, we now introduce the variable *λ*_i_, leading to the modified expression

(5) $$\varphi_{\text{IPVVB}}=\sum_{i=1}^{N}\left[\exp\left(i\left(\frac{\pi \left(x^{2}+y^{2}\right)}{\lambda_{i}f}+l\varphi +ux^{a}y^{b}-\frac{2\pi \sqrt{x^{2}+y^{2}}}{d}\right)\right)+\exp\left(-i\left(\frac{\pi \left(x^{2}+y^{2}\right)}{\lambda_{i}f}+l\varphi +ux^{a}y^{b}-\frac{2\pi \sqrt{x^{2}+y^{2}}}{d}\right)\right)\right],$$

where *N* represents the number of manipulated wavelengths. In this study, we focus on three wavelengths—780 nm, 795 nm, and 808 nm—investigating the electric field distributions of both triangular and quadrilateral perfect vector vortex beams in the x–y plane and the y–z propagation plane at the focal distance *f* = 200 μm. The experimental results are shown in Figure 5. The metasurface has the structural size of 180 μm with the numerical aperture of NA = 0.5, which is suitable for subsequent application studies. For the triangular perfect vector vortex beams, we select a superposition of RCP and LCP beams carrying topological charges of 0 and 2, denoted as $|\text{IPV}B_{R},0\rangle$ and $|\text{IPV}B_{L},2\rangle$, respectively. Meanwhile, for the quadrilateral perfect vector vortex beams, we employed RCP and LCP beams with topological charges of −1 and 7, denoted as $|\text{IPV}B_{R},-1\rangle$ and $|\text{IPV}B_{L},7\rangle$, respectively. Figure 5a–f present the multiwavelength electric field intensity distributions of the triangular beams in the x–y plane, whereas Figure 5g–l show the corresponding results for the quadrilateral beams. The first column displays the total field distributions, and the second column shows the results after passing through an analyzer. The ring size of the IPVVBs is wavelength dependent; however, due to the small wavelength difference of approximately 15 nm used in this study, the ring diameters remain virtually unchanged. Furthermore, the experimental results indicate that the topological charges and polarization orders of the rings remain consistent across different wavelengths. For the triangular beams, after passing through the analyzer, it is found that the light beam is divided into three petals, corresponding to the polarization order *p* = 1.5. In contrast, the quadrilateral beams display eight petals after passing through the analyzer, corresponding to the polarization order of *p* = 4. This further confirms that variations in wavelength do not alter the spatial polarization distribution of the beams but only affect the ring diameter. Figure 5m–p show the corresponding propagation field distributions in the y‐z plane for the triangular beams, whereas Figure 5q–s present the corresponding results for the quadrilateral beams. It can be observed that the transmission field distributions of the IPVVBs exhibit a high degree of consistency. In the near‐field region, after passing through the metasurface, the Gaussian beam initially propagates as the Bessel beam before evolving into the perfect vector vortex beam at the focal plane (*z* = 200 μm). Furthermore, as the beam continues to propagate beyond the focal plane, the ring diameter gradually increases whereas the intensity progressively decreases.

**FIGURE 6:**
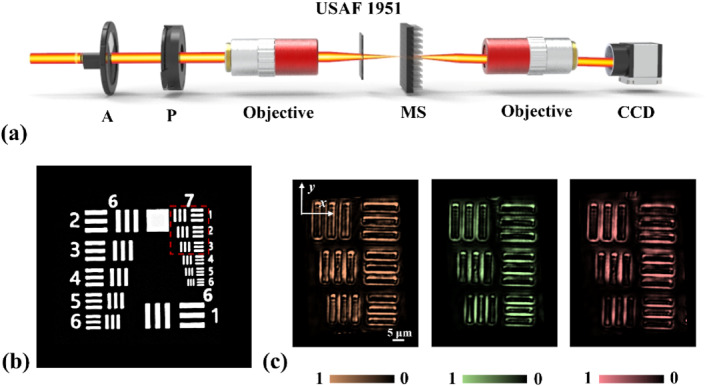
Edge imaging application. (a) Experimental setup for edge imaging. (b) USAF 1951 resolution target. (c) Edge imaging results of triangular perfect vector vortex beams at wavelengths of 780, 795, and 808 nm.

Due to the orbital angular momentum carried by irregularly shaped perfect vortex beams, the optical field exhibits a bright annular profile with a dark center. This feature enables the pronounced response to object edges. In addition, the vector beam forms a strong transverse field component when it is focused, making the interaction between the beam and the object boundary more significant. Based on the above multiwavelength multiplexing results, we further demonstrate the corresponding edge imaging application, as illustrated in Figure 6. Figure 6a illustrates the experimental setup for edge imaging. An incident Gaussian beam passes through a filter and then a polarizer (P) to generate the required linearly polarized light. The beam is subsequently focused by an objective lens onto USAF 1951 resolution target. The transmitted light illuminates the metasurface, generating the target‐shaped perfect vector vortex beam. This output beam is then collected by a second objective lens and imaged by the CCD camera. Figure 6b displays Groups 6 and 7 of the DaHeng Optics USAF 1951 resolution target. Edge imaging is performed on Elements 1, 2, and 3 of Group 7. Firstly, edge imaging is performed using the triangular perfect vector vortex beams generated in Figure 5. The corresponding imaging results at wavelengths of 780 nm, 795 nm, and 808 nm are shown in Figure 6c, with the highest resolution reaching 3.1 μm. This application experiment lays a foundation for potential uses in fields such as microscopic imaging and biomedicine.

## 3 Conclusion

In summary, we have proposed the metasurface capable of generating IPVVBs and demonstrated both theoretically and experimentally the ability to achieve arbitrary polarization control of vector beams on the hybrid Poincaré sphere. By introducing cross‐phase modulation, we achieve precise control over the beam shape. The superposition of left‐hand and right‐hand beams with different topological charges enables the generation of integers and fractional order triangular and quadrilateral perfect vector vortex beams. Additionally, we have demonstrated successful polarization control at the positions (0, π/2), (0, π), and (π/6, 0) on the Poincaré sphere. Furthermore, we investigate triangular and quadrilateral perfect vector vortex beams at wavelengths of 780 nm, 795 nm, and 808 nm, demonstrating the capability for wavelength multiplexing. Finally, using the triangular vector vortex beam with polarization order *p* = 1.5, we perform edge imaging of Elements 1, 2, and 3 in Group 7 of the USAF 1951 resolution target, achieving a resolution of 3.1 μm. We believe that this work provides a novel strategy for the generation of arbitrarily shaped vector structured optical fields and will make significant progress in the fields of biomedical imaging, materials inspection, and optical information processing.

## Supporting information


Supporting Information S1

## Data Availability

The data that support the findings of this study are available from the corresponding author upon reasonable request.
