# Supplementary material for: The Generation and Control of Irregularly Shaped Perfect Vector Vortex Beams on Hybrid Poincaré Spheres Using All‐Dielectric Metasurfaces
Source: Nanophotonics. 2026 Jan 29;15(3):e70020. doi: 10.1002/nap2.70020 (PMC12965007; doi:10.1002/nap2.70020)
Supplement: Supplementary file 1 — Supporting Information S1 [file NAP2-15-e70020-s001.docx]

Supporting Information

**The generation and control of irregularly shaped perfect vector vortex beams on hybrid Poincaré spheres using all-dielectric metasurfaces**

Xiaojie Sun, Xuan Yang, Haixu Tao, Haiyang Song, Jingpei Cai, Jiaxuan Zou, Jiajing He*, and Jun Wang*

**Supplementary Section 1: Theoretical Analysis of Generating IPVVB**

The metasurface designed for generating Irregular-shaped Pefect Vector Vortex Beams is constructed from an array of anisotropic rectangular nanounits. Under incidence of linearly polarized light on the metasurface structure, the transmitted beam retains the same polarization as the incident light; therefore, the Jones vector of the transmitted beam can be expressed as:

$$\left[\begin{matrix} 1 \\ 0 \end{matrix}\right]=\frac{1}{\sqrt{2}}\left(\frac{1}{\sqrt{2}}\left[\begin{matrix} 1 \\ i \end{matrix}\right]+\frac{1}{\sqrt{2}}\left[\begin{matrix} 1 \\ -i \end{matrix}\right]\right)$$

To generating IPVVB, the linearly polarized beam can be decomposed into the superpositon of right- and left-circular polarization components, as follows:

where *l_m_* and *l_n_* denote the orbital angular momenta of the right-hand and left-hand circular polarized vortex beams, respectively. After simplification, we obtain:

Here, the first and second terms represent the phases of the left- and right-ciucularly polarized components, respectively, while $\varphi(x,y)$ denotes the target phase. Therefore, the utilization of geometric phase in the proposed approach enables the generation and polarization control of arbitrary IPVVB on the hybrid Poincaré sphere.

**Supplementary Section 2: Unit Cell Design and Polarization Conversion Efficiency of the Structure**


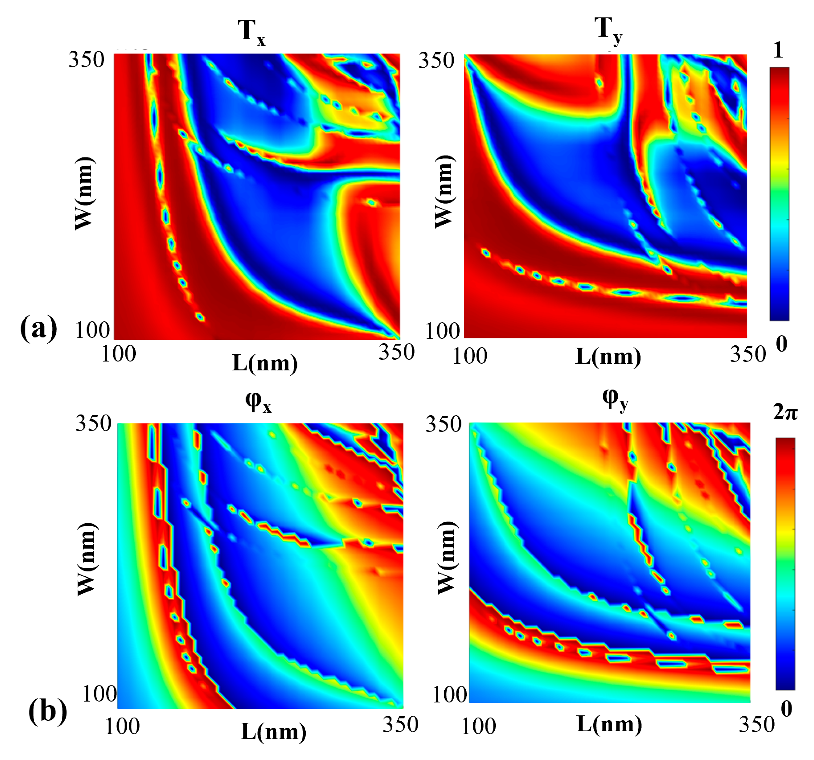


**Figure S1.** Parameter sweep results of the amorphous silicon nanounit structure at a wavelength of 808 nm. (a) Transmittance (*T_x_* and *T_y_*) versus the nanounit size parameters, length *L* and width *W*. (b) Phase ($\varphi_{x}$ and $\varphi_{y}$) versus the nanounit size parameters, length *L* and width *W*.


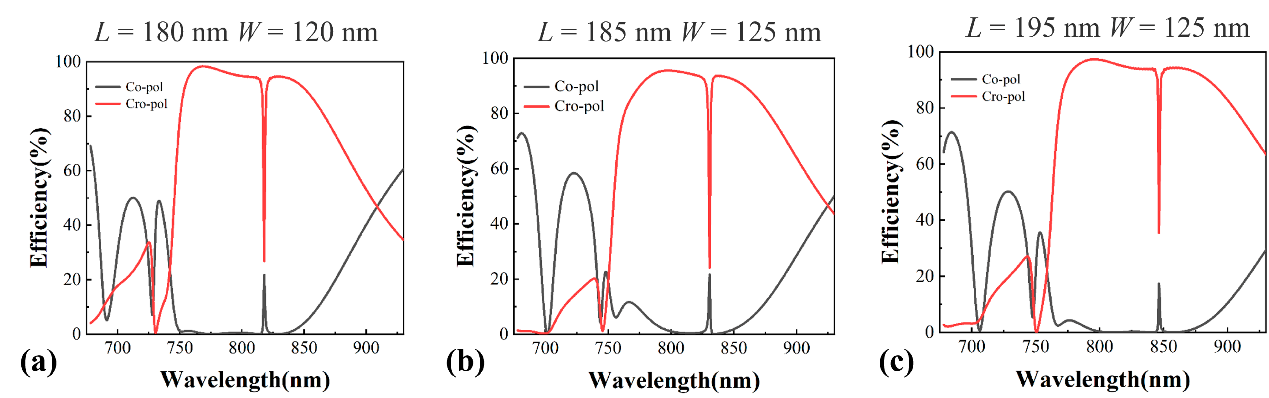


**Figure S2.** Polarization conversion efficiency for different structural dimensions at a wavelength of 808 nm. (a) Polarization conversion efficiency of the structure with *L* = 180 nm, *W* = 120 nm. (b) Polarization conversion efficiency of the structure with *L* = 185 nm, *W* = 125 nm. (c) Polarization conversion efficiency of the structure with *L* = 195 nm, *W* = 125 nm.

**Supplementary Section 3: Vector Rayleigh–Sommerfeld Diffraction Theory**

In the main text, the numerical results were simulated using the Rayleigh–Sommerfeld diffraction theory, with the metasurface size set to 72 μm and the far-field focal length to *f* = 200 μm. Here, the vector Rayleigh–Sommerfeld diffraction theory is written as:

$$E\left(x,y\right)=\frac{e^{ikz}}{i\lambda z}\iint E\left(u,v\right)\frac{e^{ik\rho}}{\rho}dxdy$$

where *k* is the wave vector, $\lambda$ is the wavelength, and E(*u,v*) represents the complex amplitudes of the IPVVB at z = 0. $\rho=\sqrt{\left(x-u\right)^{2}+\left(y-v\right)^{2}+z^{2}}$.

**Supplementary Section 4:** **Intensity Distributions of IPVVB Under Different Incident and Analyzer Polarizations**


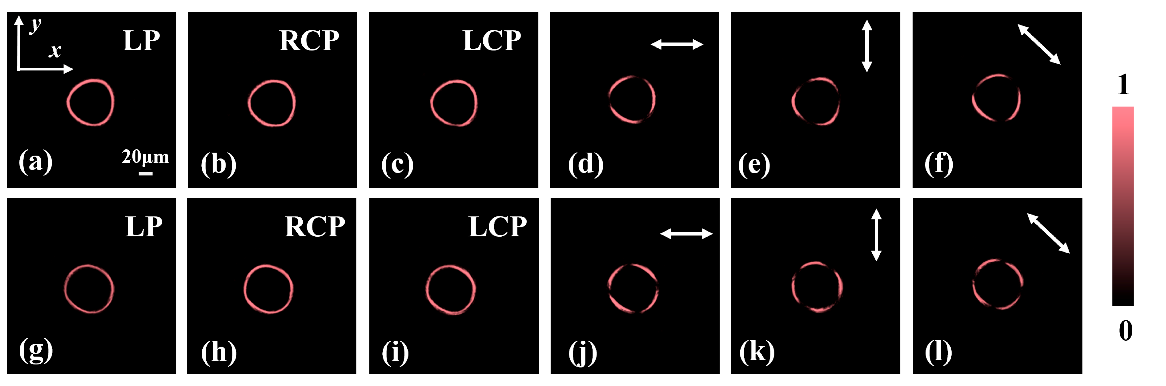


**Figure S3.** Experimental intensity distributions of IPVVB for different incidence and analyzer polarizations. (a)-(f) Intensity distributions of the triangular perfect vector vortex beams: (a)-(c), total field distributions under linearly polarized, right-circularly polarized, and left-circularly polarized incidence, respectively; (d)-(f), intensity distributions under a linearly polarized incident Gaussian beam through analyzers at 0°, 90°, and 135°, respectively. (g)-(i) Intensity distributions of the quadrilateral perfect vector vortex beams under the same conditions as (a)–(f).

**Supplementary Section 5:** **Optical Field Manipulation of IPVVB with Varying Parameters**


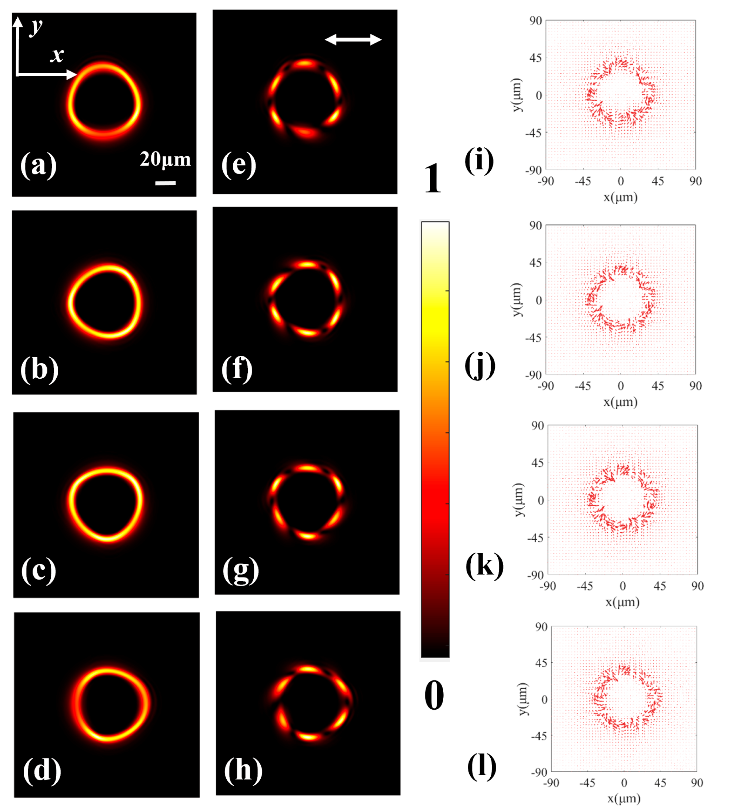


**Figure S4.** Intensity distributions of triangular perfect vector vortex beams with polarization order *p* = 3 for different combinations of a and b; the cross-phase parameter is set to *u* = 1.5 × 10^14^. (a)-(d) Total field distributions for (*a, b*) = (0, 3); (1, 2); (2, 1); (3, 0), respectively. (e)-(h) Intensity distributions of the triangular perfect vector vortex beams after transmission through analyzers. (i)–(l) Electric field vector distributions of the corresponding triangular perfect vector vortex beams.


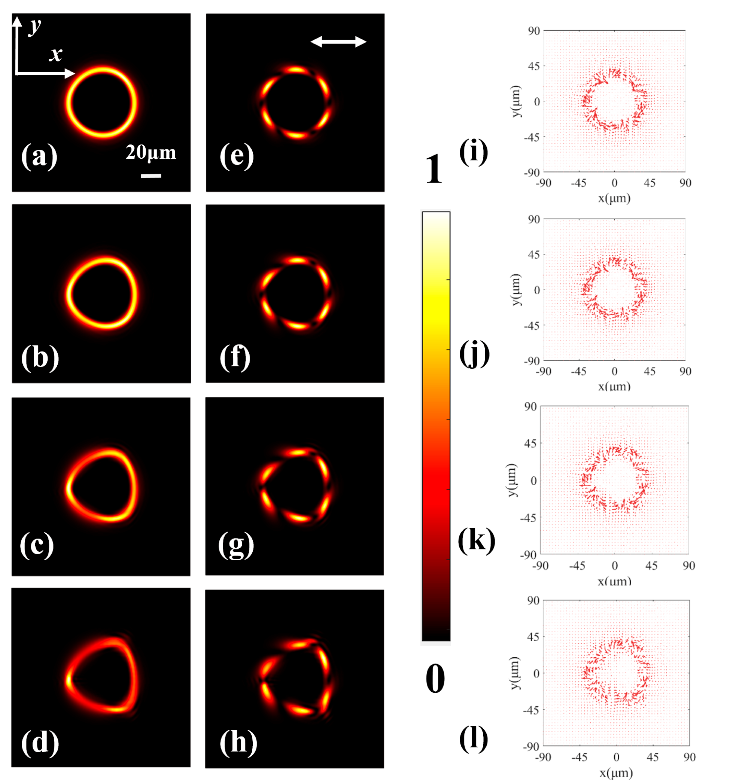


**Figure S5.** Under the cross-phase condition with *a* = 1 and *b* = 2, the intensity distributions of triangular perfect vector vortex beams with polarization order *p* = 3 are investigated for different values of *u*. (a)–(d) show the total field distributions for *u* = 0.5 × 10^14^, *u* = 1.5×10^14^, *u* = 3×10^14^, and *u* = 5×10^14^ respectively. (e)–(h) display the corresponding intensity distributions after passing through an analyzer. (i)–(l) present the corresponding electric field vector distributions of the triangular perfect vector vortex beams.

**Supplementary Section 6: Fabrication Process**


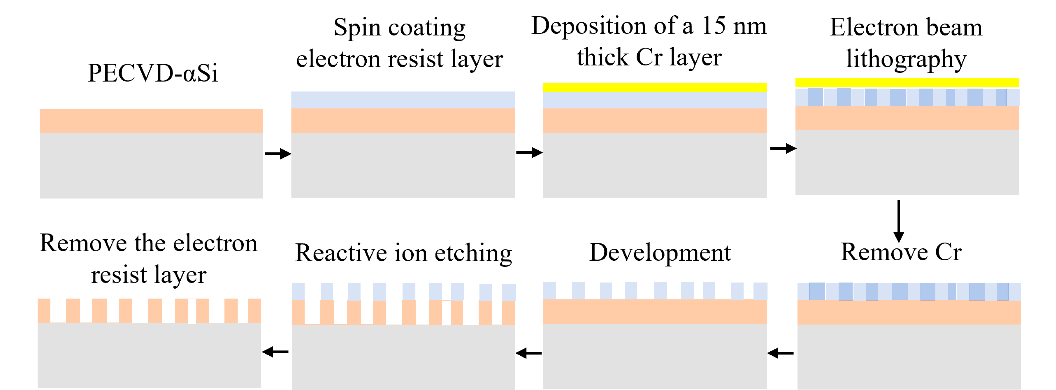


**Figure S6.** Fabrication steps

We first deposit a 600 nm αSi on a SiO_2_ substrate by PECVD (Plasma Enhanced Chemical Vapor Deposition). A 180 nm-thick layer of CASR62 resist was spin-coated on the samples at 4000 rpm for 60 s. Then, the wafers were baked at 180 ℃ for 90 s. After that, a 15-nm of Cr was deposited on the ebeam resist by electron beam evaporator. The CASR62 resist was exposed by electron beam lithography (Vistec EBPG5200). After the deposition, the Cr was then removed and developed in MIBK. CSAR62 is a positive electron beam resist. After that, the patterns were transferred to the SiO_2_ device layer by ICP-RIE Deep Silicon Etching System (SPTS).

**Supplementary Section 7: Normalization of light intensity contrast analysis**


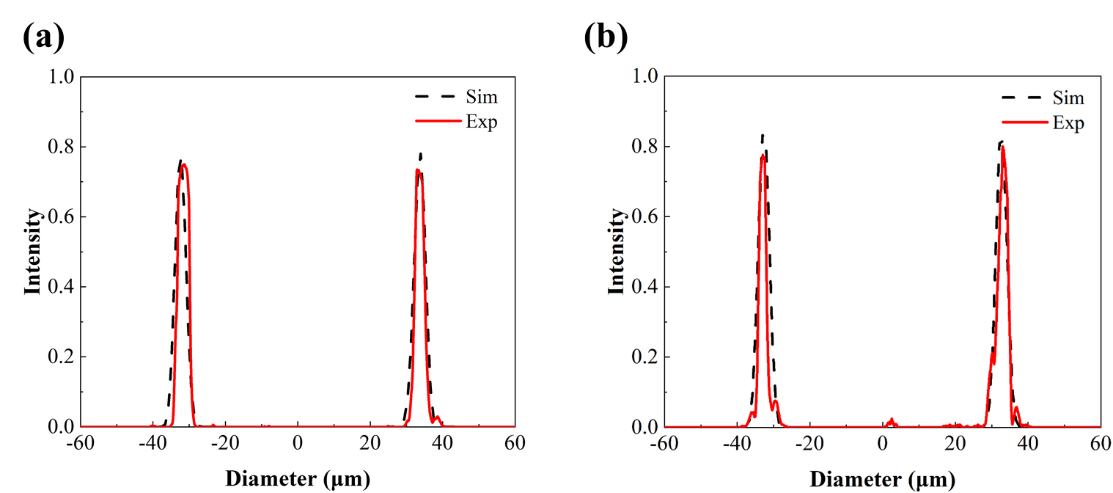


**Figure S7.** Comparison analysis of the normalized intensity of perfect vector vortex beams in triangles and quadrilaterals in experiments and simulations

**Supplementary Section 8: The influence of parameter d**


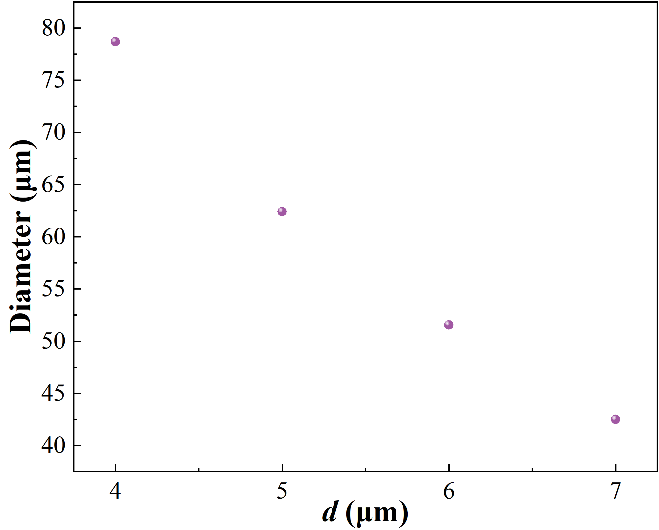


**Figure S8.** The variation of ring diameter with different parameters d.
